# Migraine Increases Centre-Surround Suppression for Drifting Visual Stimuli

**DOI:** 10.1371/journal.pone.0018211

**Published:** 2011-04-11

**Authors:** Josephine Battista, David R. Badcock, Allison M. McKendrick

**Affiliations:** 1 Department of Optometry & Vision Sciences, The University of Melbourne, Parkville, Australia; 2 School of Psychology, The University of Western Australia, Nedlands, Australia; Istituto di Neuroscienze, Italy

## Abstract

**Background:**

The pathophysiology of migraine is incompletely understood, but evidence points to hyper-responsivity of cortical neurons being a key feature. The basis of hyper-responsiveness is not clear, with an excitability imbalance potentially arising from either reduced inhibition or increased excitation. In this study, we measure centre-surround contrast suppression in people with migraine as a perceptual analogue of the interplay between inhibition and excitation in cortical areas responsible for vision. We predicted that reduced inhibitory function in migraine would reduce perceptual surround suppression. Recent models of neuronal surround suppression incorporate excitatory feedback that drives surround inhibition. Consequently, an increase in excitation predicts an increase in perceptual surround suppression.

**Methods and Findings:**

Twenty-six people with migraine and twenty approximately age- and gender-matched non-headache controls participated. The perceived contrast of a central sinusoidal grating patch (4 c/deg stationary grating, or 2 c/deg drifting at 2 deg/sec, 40% contrast) was measured in the presence and absence of a 95% contrast annular grating (same orientation, spatial frequency, and drift rate). For the static grating, similar surround suppression strength was present in control and migraine groups with the presence of the surround resulting in the central patch appearing to be 72% and 65% of its true contrast for control and migraine groups respectively (t(44) = 0.81, p = 0.42). For the drifting stimulus, the migraine group showed significantly increased surround suppression (t(44) = 2.86, p<0.01), with perceived contrast being on average 53% of actual contrast for the migraine group and 68% for non-headache controls.

**Conclusions:**

In between migraines, when asymptomatic, visual surround suppression for drifting stimuli is greater in individuals with migraine than in controls. The data provides evidence for a behaviourally measurable imbalance in inhibitory and excitatory visual processes in migraine and is incompatible with a simple model of reduced cortical inhibitory function within the visual system.

## Introduction

Migraine is an episodic neurovascular brain disorder that causes significant burden to both individuals and society [1]. Prevalence estimates vary, however, typically are around 15% of the adult population [2], [3]. Migraine is diagnosed based on symptomatology [4] as no diagnostic objective tests for migraine are currently available.

Visual symptoms are common in migraine either in the form of aura, or photophobia, or less specific symptoms such as blur and accommodative dysfunction [4]. Because the visual pathways are clearly implicated at least during the acute migraine event, visual perception has been extensively used as a method to indirectly explore brain function in migraine (for example: [5], [6], [7], [8], [9]). The majority of visual processing studies in migraine have tested participants between migraine events (for example: [5], [6], [7], [8], [9]), and have provided evidence for differences in brain function. These differences can be broadly characterized into two types: a) threshold deficits in performance (for example: reduced contrast sensitivity [10], [11], visual field loss [12], [13], [14], [15], elevated motion coherence thresholds [7], [8], [9]; and b) perceptual differences for suprathreshold stimuli such as increased aversiveness to striped patterns [16], or differences in adaptational status [5], [17], [18].

Migraine pathogenesis is incompletely understood, however, most current models of the disease process invoke a mechanism of brain hyperresponsivity (also referred to as hyperexcitability) [19], [20], [21], [22]. These terms have been used interchangeably in the literature, and are not always clearly defined in terms of a proposed neural basis. Simplistically, hyperexcitable neurons might respond to stimuli that would otherwise be subthreshold, however there is little evidence for this in migraine. For example, most psychophysical studies show elevated thresholds rather than hypersensitivity of threshold responses (for example see: [7], [8], [9], [10]). Alternately, hyperexcitability might refer to increased spontaneous neural firing therefore elevated neural noise. This argument has been used to explain elevated threshold responses on motion coherence tasks where threshold performance relies on the detection of signal from noise [23], and elevated thresholds when visual noise is added to luminance targets [24]. It has also been proposed that neuronal hyperresponsivity could arise as a secondary manifestation of reduced cortical inhibition from downregulated GABA-ergic activity [25], [26], [27] or that the cortex has reduced cortical preactivitation levels possibly due to serotonergic hypoactivity [28].

A key aspect of visual processing that requires a balance of inhibitory and excitatory neural networks is the modulation of neuronal responses depending on the surrounding context. Visual neurons, at various stages of the visual system, respond to stimuli presented within their classical receptive field, however, the magnitude of the response is regulated depending on the surrounding image features [29], [30]. The neural systems regulating the effect of the surround on the classical receptive field (CRF) are complex, but are understood to involve both feedforward responses from earlier sites in the visual pathways, feedback responses from extrastriate visual cortices, as well as lateral inhibition within primary visual cortex (for review see: [31], [32]).

In this study, we explore the balance between inhibition and excitation in migraine by measuring visual performance for a well-studied perceptual analogue of centre-surround neuronal responses: the Chubb illusion (also referred to as contrast-contrast suppression) [33]. The most common versions of the task involve measuring the perceived contrast of a patch of sinusoidal grating in the presence of an annular surround [33], [34], [35], [36], [37]. If the surround is of higher contrast and of like-orientation to that of the centre, the perceived contrast of the centre patch will be reduced [35], [36], [37]. Depending on a range of stimulus attributes (such as orientation, phase, spatial frequency, size of annulus), the magnitude of the suppression can be modulated [35], [36], [37]. For this study, we chose a sub-set of experimental conditions that elicit a substantial shift in perceived contrast in normal observers (approximately a 20–40% reduction of apparent contrast), and measured the magnitude of contrast-contrast surround suppression for both a stationary and a drifting grating version of the stimulus. Most studies of contrast-contrast suppression have used stationary stimuli, however, we included a drifting version as previous work demonstrates deficits in the processing of visual motion in people with migraine [7], [17], [23], [38]. Spatial pattern information and motion information are processed in the separate, but intercommunicating, ventral and dorsal visual substreams respectively, hence the drifting and stationary tasks will preferentially bias detection to different neural pathways [39], [40].

We hypothesized that reduced cortical inhibition in migraine would result in decreased perceptual surround suppression on the contrast-contrast task, whereas, a general increased level of neural excitation would increase the effect of the surround on central responses. This seemingly counterintuitive prediction of an increase in suppression from elevated neural excitability results from models of surround suppression that incorporate contrast dependent feedback excitation to drive lateral inhibition [31], [32]. A generalised dysfunction of cortical areas responsible for vision predicts altered performance for both static and drifting stimulus versions. Our experimental results show an increased strength of perceptual surround suppression in the migraine group for the drifting stimulus only.

## Methods

### Ethics

Ethics approval for the project was obtained from the Human Research Ethics Committee of the University of Melbourne. Prior to participation, written informed consent was provided in accordance with a protocol approved by our institutional human research ethics committee and in accordance with the tenets of the Declaration of Helsinki.

### Participants

The migraine group consisted of 26 participants: 12 fulfilling the International Headache Society's [4] criteria for migraine with aura (MA) and 14 the criteria for migraine without aura (MO). Twenty approximately age- and gender-matched controls also participated. For inclusion in the control group, participants were required to experience fewer than 4 headaches per year, and to have never experienced a headache or migraine which fulfilled the International Headache Society criteria [4]. Participants in the migraine group (23 female, 3 male) were aged between 18 and 44 years (mean = 32 ± SD = 6 years), and control participants (15 female, 5 male) were aged from 24 to 41 years (mean = 29 ± SD = 5 years). There was no significant difference in mean age (*t*_(44)_ = 1.70, *p* = 0.10) or gender (χ^2^(1, *N* = 46) = 1.43, *p* = 0.23) between these groups.

All participants were required to have best corrected visual acuity of 6/7.5 or better and to have refractive errors less than ±5.00 D sphere and ±2.00 D astigmatism. Participants were required to be free from systemic disease known to affect visual or cortical function and were also not permitted to be taking medications known to affect visual or cortical function. Normal findings in a comprehensive eye examination conducted as part of the study were also required. This examination included slit lamp biomicroscopy, ophthalmoscopy of the macula and optic nerve and an intraocular pressure measurement with applanation tonometry (less than 21 mm Hg was required for inclusion).

Prophylactic medications for migraine were not permitted and participants were tested at least 4 days since the end of their last migraine in order to allow recovery from the episode and washout of any medications taken to relieve migraine symptoms. Participants in the migraine group completed the MIDAS (migraine disability assessment) questionnaire to enable a basic measure on the current impact of migraine on their lives by scoring the impact of headaches over the past 3-months on tasks of daily living [41]. MIDAS scores are typically interpreted as follows: grade 1, minimal or infrequent disability (score 0–5); grade 2, mild disability (score 6–10); moderate disability (score 11–20), and severe disability (score 21+). The migraine participants of this study had MIDAS scores between zero and 95 (mean = 23 ± SD = 20).

### Procedures

Stimuli were presented on a gamma-corrected, 21-inch monitor (resolution: 800×600 pixels; frame rate: 120 Hz; G520 Trinitron; Sony, Tokyo, Japan) and generated with a ViSaGe system (Cambridge Research Systems, Ltd., Kent, UK) using custom software written in Matlab 7 (Mathworks, Natick, Massachusetts). Participants indicated responses using a button box (model CB6; Cambridge Research Systems) and viewed the monitor binocularly from a distance of 100 cm using a chin and forehead rest, with their required refractive correction for the viewing distance.

**Figure 1:**
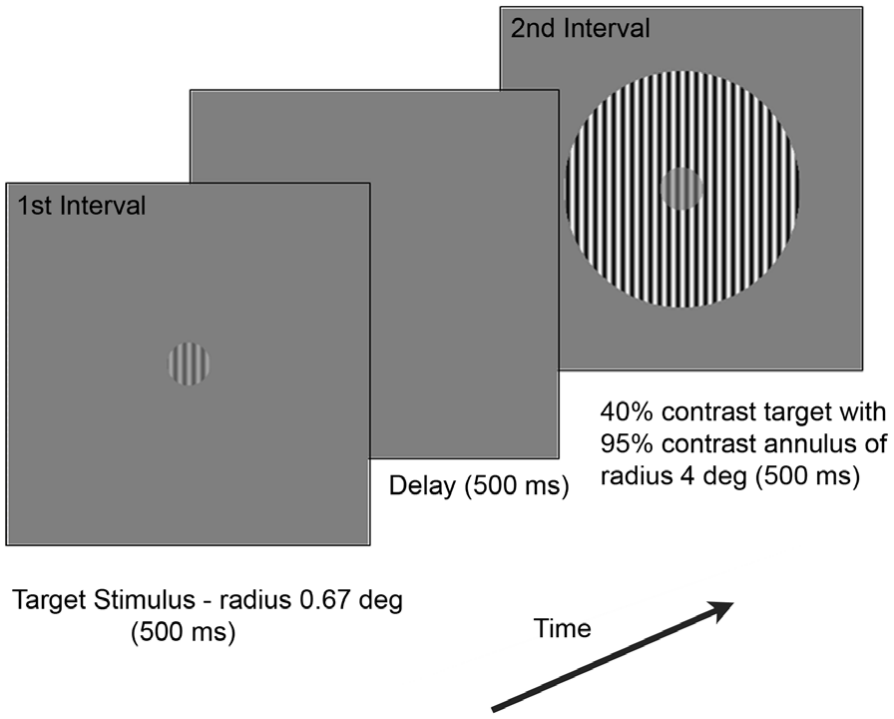
Schematic representation of the centre-surround contrast discrimination task. Consecutive stimuli were presented to participants who indicated which of the two intervals had the centre stimulus of higher contrast. The target stimulus (front panel) was presented for 500 ms followed by a 500 ms inter-stimulus interval. A second stimulus was then displayed for 500 ms and was comprised of either the target stimulus alone (no-surround condition) or surrounded by an annulus of 95% contrast with a radius of 4 degrees (surround condition – back panel). The smaller centre stimulus had a radius of 0.67 degrees and seven different contrast levels were randomly presented.

The static and motion tasks were investigated in separate runs, the order of which was randomized between participants. Participants completed trials within a session of approximately 2 hours in duration, with practice trials to familiarize themselves with the task and rest breaks permitted as required. A schematic representation of the stimuli used for the experiments is shown in Figure 1. The central target grating had a radius of 0.67 degrees. For the static task, the grating had a spatial frequency of 4 c/deg, while for the motion task, the grating had a spatial frequency of 2 c/deg, drifting at a rate of 2 deg/sec (the centre and surround targets moved together – contrast border only). When the target had a surround, it was embedded in a 4 degrees radius annulus of 95% contrast, with the same phase, orientation and spatial frequency as the centre.

Using a two-interval forced choice paradigm, participants performed two contrast discrimination tasks for the static and moving stimuli and had to choose which interval had the stimulus with the highest contrast. In the *‘no-surround’* condition, participants discriminated a difference in contrast between two central target gratings. For the *‘surround’* condition, it was determined how performance changed when the target was presented within a surrounding annulus (see back panel of Figure 1). For the ‘no-surround’ condition, the first interval consisted of the target grating presented at a variable contrast for 500 ms. After an inter-stimulus interval of 500 ms, a second patch of a fixed contrast of 40% was presented, also for 500 ms. Observers were instructed to indicate in which of the two intervals they perceived the target stimulus as having the higher contrast. The contrast of the patch presented in the first interval was varied to enable a psychometric function to be obtained using a method of constant stimuli (MOCS). Seven contrast levels were randomly interleaved and were each presented 20 times. Each participant initially performed an abbreviated MOCS (13 levels, presented 4 times each) that was used to select the range of the 7 contrasts used in the final MOCS procedure. This same procedure was used for the “surround” condition, except that in this case, the 40% contrast target patch presented in the second interval was surrounded by a high contrast annulus (as shown in Figure 1).

### Data analysis and statistics

**Figure 2:**
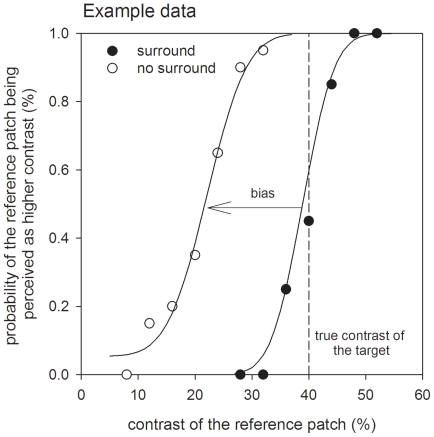
Example psychometric function for a single participant. The filled and open symbols show the raw data collected for the no surround and surround conditions respectively. Fitted curves are the best fitting cumulative Gaussian distributions to the data. The curves have similar spreads (slope of the psychometric function), however, the presence of the annular surround results in a leftwards shift of the curve (unfilled symbols) because the target patch appears to be lower contrast than the veridical contrast of 40% (dashed vertical line). Bias was determined as the shift in the point-of-subjective equality (PSE, mean of the best fitting cumulative Gaussian) caused by the annular surround.

Example results obtained from a MOCS procedure are shown in Figure 2. These sigmoidal shaped psychometric functions describe the probability of perceiving the reference patch as higher in contrast than the target patch, as a function of reference patch contrast. Figure 2 shows data for a single non-headache control participant. Raw data was fit with a modified cumulative Gaussian [42]:
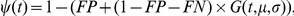
where 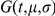 is the cumulative Gaussian with mean *μ* and standard deviation *σ* for value *t*. *FP* and *FN* represent the false positive and false negative rates respectively. This form of the psychometric function provides for *FP* and *FN* errors that are made independently of the Gaussian response distribution. Curve fitting was achieved using a bootstrap procedure [43]) of 1000 repetitions which enabled 95% confidence limits to be estimated for *μ*, *σ*, *FP* and *FN*.

The parameters of key interest for subsequent statistical comparison between groups were: a) the mean of the Gaussian *μ*, which in this study represents the contrast level for which the reference patch subjectively appears the same contrast as the target patch (the “point of subjective equality”, PSE); and b) the spread of the Gaussian *σ* which provides an estimate of contrast discrimination precision for each observer. An estimate of bias was calculated for each observer, which was determined as the [PSE for the annular condition – PSE for the target patch alone] (see Figure 2 for illustration).

Statistical analyses were performed using SPSS 16.0 (SPSS Inc. IBM Corporation, Somers, NY, USA) with t-tests or repeated measures, mixed design ANOVA analyses as appropriate. Huynh-Feldt adjustments were used for non-spherical data. No significant difference between migraine groups was demonstrated on any measure (independent sample t-tests, all *p*>0.05) therefore the results for the MA and MO groups were pooled for comparison against controls.

## Results

### Centre-surround suppression for the non-drifting stimulus

**Figure 3:**
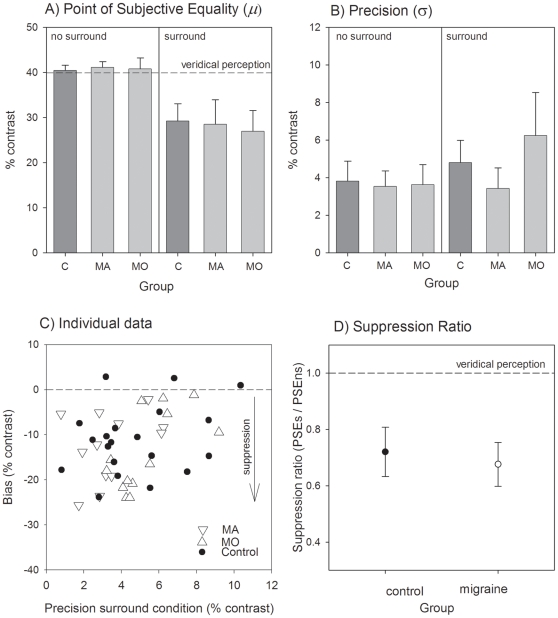
Centre-surround suppression for parallel static gratings. Panel A shows the PSE for the isolated centre patch (right hand side of panel: LHS) and when presented within the surround (left hand side of panel: RHS). Group means (±95% confidence intervals of the mean) are shown for control participants (C), migraine with aura (MA) and migraine without aura (MO) groups. Panel B shows the precision (spread of psychometric functions for the same groups, also for the no surround (LHS) and surround (RHS) conditions. Individual performance for each participant is shown in Panel C which plots the bias (shift in PSE) against their precision for the surround condition. Panel D shows the group mean (±95% confidence intervals of the mean) suppression ratio for the controls and all pooled migraine participants. The suppression ratio was determined as the PSE for the surround condition divided by that for the no surround condition. A ratio of 1 indicates that the surround has no effect. A reduction in the apparent contrast of the central patch due to the surround results in a suppression index less than 1.

Figure 3, panel A, shows the group mean PSE (±95% confidence interval for the mean) for the controls, migraine with aura (MA) and migraine without aura (MO) groups. Inspection of the left of Figure 3A shows that all groups were able to accurately perform the contrast discrimination task for the no-surround condition as the PSE was close to the veridical contrast of 40%. When the surround was present, all groups experienced significant surround suppression with the mean PSE being less than 30% contrast in all groups. A RM-ANOVA (within factor: surround or no-surround condition; between factor: migraine or control group: note that the migraine groups were combined) showed that the group means were not significantly different (F(1,44) = 0.15, p = 0.70), and that there was no significant interaction between group and condition (F(1, 44) = 0.80, p = 0.37).

Figure 3B shows group mean precision in performing the task (spread of the psychometric function). There was no significant difference between groups for this measure (F(1,44) = 0.005, p = 0.94), however, both migraine and control participants showed reduced precision (flatter psychometric functions) when the annulus was present (main effect of condition: F(1,44) = 6.0, p = 0.01; no significant interaction between group and condition (F(1,44) = 0.16, p = 0.69)).

All individual data is shown in Figure 3C which plots the bias (PSE surround – PSE no surround) against precision for the surround condition. Consistent with the group mean performance analysis, inspection of Figure 3C reveals near complete overlap in the range of scores within the migraine and control groups.

Panel 3D shows the group mean suppression ratio (PSE surround divided by PSE no surround) and 95% confidence intervals of the mean. The presence of the surround resulted in the target patch appearing to be 72% and 65% of its true contrast for the control and migraine groups respectively and these values were not significantly different (t(44) = 0.81, p = 0.42).

### Centre-surround suppression for the drifting stimulus

**Figure 4:**
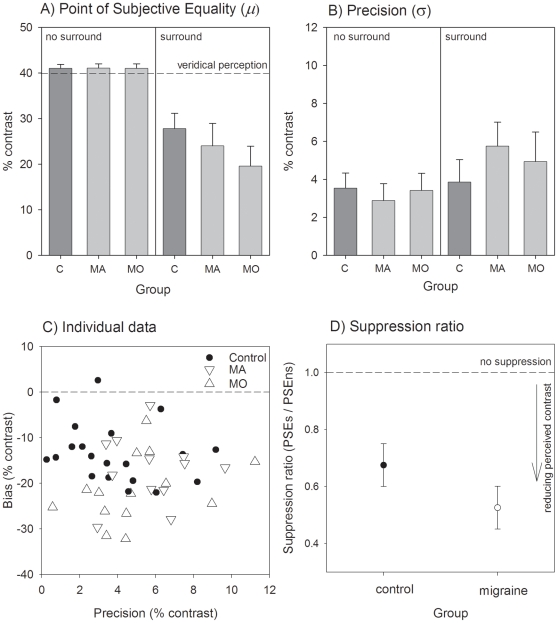
Centre surround suppression for parallel drifting gratings. Panel A shows the PSE for the isolated centre patch (right hand side of panel: LHS) and when presented within the surround (left hand side of panel: RHS). Group means (±95% confidence intervals of the mean) are shown for control participants (C), migraine with aura (MA) and migraine without aura (MO) groups. Panel B shows the precision (spread of psychometric functions for the same groups, also for the no surround (LHS) and surround (RHS) conditions. Individual performance for each participant is shown in Panel C which plots the bias (shift in PSE) against their precision for the surround condition. Panel D shows the group mean (±95% confidence intervals of the mean) suppression ratio for the controls and all pooled migraine participants. The suppression ratio was determined as the PSE for the surround condition divided by that for the no surround condition. A ratio of 1 indicates that the surround has no effect. A reduction in the apparent contrast of the central patch due to the surround results in a suppression index less than 1.

Figure 4 presents data for the drifting stimulus condition in the same manner as in Figure 3. For the drifting task, all groups were similarly able to perform the contrast discrimination task when the annulus was not present (inspection of the left hand side of Fig. 4A and Fig. 4B). However, the presence of the surround caused a greater reduction in perceived contrast for the migraine participants than for the controls (right side of Fig. 4A and suppression ratios in Fig. 4D). A RM-ANOVA comparing the pooled migraine group to controls showed significant main effects of group (F(1,44) = 6.0, p = 0.02) and condition (F(1,44) = 234, p<0.001). The interaction between group and condition was significant (F(1,44) = 8.40, p<0.01) confirming the differential effect on perceived contrast between groups for the surround condition relative to the no-surround condition. Eight migraine participants (6 MO and 2 MA) demonstrated more surround suppression than the maximally suppressing control individual (Figure 4C). The presence of the surround resulted in the perceived contrast being 68% of actual contrast for the control group, and 53% of actual contrast for the migraine group (t(44) = 2.86, p<0.01; data shown in Figure 4D). There was no statistically significant main effect of group on precision in performing the task (F(1,44) = 1.24, p = 0.27: see Figure 4B).

### Relationship to headache features

For the migraine participants, we examined whether the shift in PSE (PSE surround – PSE no surround) correlated with any of the following migraine characteristics: MIDAS score, frequency of migraines, time since last migraine, and age at first migraine. None of these correlations were significant (*p*>0.05).

## Discussion

The strength of perceptual centre-surround processing was increased in the migraine group relative to non-headache controls for drifting stimuli. However, for stationary stimuli, centre-surround effects were similar in magnitude between groups. To our knowledge, the only other published paper that reports on surround suppression in migraine is from our own laboratory [38]. In that study, direction discrimination duration thresholds were measured for briefly presented drifting grating stimuli of varying sizes and contrasts (as per the methods described in [44]). In that study, a subtle alteration in surround suppression in migraine was observed. The current paper supports and extends the previous work by showing a far more robust relative increase in surround suppression in the migraine group for the contrast-contrast task, and by demonstrating a specific alteration in the processing of drifting but not static stimuli.

We found no relationship between the strength of surround suppression and specific migraine features, including the presence or absence of visual aura. A number of other studies have similarly been unable to distinguish aura and non-aura groups between their attacks on other psychophysical measures (for example: [6], [8], [45] [46], [47]), as may be expected if performance differences arise from brain anomalies that relate to susceptibility to migraine rather than processes occurring during the actual migraine events. It should be kept in mind however that our migraine group was a community sample hence reflects the mild end of migraine severity spectrum, in comparison to a tertiary neurology patient base.

Our findings are not consistent with a hypothesis of generally reduced cortical inhibitory function in migraine because a simple model of reduced inhibition would predict reduced contrast-contrast suppression. Reduced cortical inhibition has been used by others as an explanation for reduced perceptual surround suppression measured in schizophrenia [48], [49] and in the elderly ([50], but see also [51]). Instead, our findings can potentially be explained by a model of enhanced excitatory feedback increasing the strength of lateral inhibition. The neurophysiology of surround suppression is an area of intense research interest at present, and as knowledge evolves regarding the interplay between the complex networks of feedforward, feedback and lateral connections, our data may require reinterpretation.

We expected performance differences between groups for both the drifting and non-drifting stimuli because a general alteration in brain responsiveness doesn't lend itself to a simple prediction of enhanced susceptibility to a particular stimulus subset. However, a clear separation between migraine and control group performance was only measurable for the drifting stimulus. Flickering and moving stimuli have been previously shown to be useful in identifying differences between migraine and non-migraine groups [7], [17], [23], [38], [52], [53], [54]. Initial motivation for the use of flickering stimuli presumably stemmed from symptomatic reports of aversion to flicker in migraine groups, and also reports of flickering visual conditions contributing to the onset of migraine events (for review see: [55]). The cause of anomalous processing of flickering stimuli in migraine is not clear. There is evidence that flickering stimuli result in increased blood flow within the retina and optic nerve [56] and cortically [57]. If migraine disrupts such neuro-vascular coupling then abnormalities in function may arise that relate in magnitude to flicker rate. However, it is not clear why this would drive increased surround suppression. Furthermore, flickering stimuli elicit maximal vascular response change for frequencies around 15 Hz [57] which is substantially higher than the temporal information in our stimulus.

An alternate explanation for motion specific deficits in migraine is specific involvement of the extrastriate visual areas responsible for motion processing (areas V5 and V3a). Transcranial magnetic stimulation responses in V5 are different compared to non-headache controls [58]. Migraine groups have elevated motion coherence thresholds [7], [8], [23] for random-dot-motion stimuli that require global integrative mechanisms such as those present in V5 [59] Structural increases in cortical thickness in areas V5 and V3a have been reported in individuals with migraine as well as subtle white-matter changes in the superior colliculus revealed with diffusion tensor imaging [60]. The dorsal pathway from V1 through to V5 is well studied, however a less-well understood pathway to V5 involves considerable feedforward projections from the superior colliculus to area MT [61]. As the brainstem has been proposed to have a key role in migraine pathogenesis ([62] [63]), aberrant brainstem input could also produce altered function in cortical visual motion areas.

An alternate consideration is whether the pattern of results is explicable by a non-visual mechanism such as differences in attention, aversion or some other non-visual difficulty in performing the drifting relative to the static task. Previous research shows that migraine groups find high contrast patterns of between 2–4 c/deg more aversive than non-headache controls [16], [47]. Because precision in performing the task was similar between groups and tasks (see Figures 3B and 4B) a stimulus-specific aversion explanation for the data seems unlikely. We did not formally measure aversion, however, the examiner informally questioned the participants regarding the testing after completion and did not receive regular reports of task discomfort.

Perceptual performance measures in people with migraine have the potential to inform about differences in neural activity in the migrainous brain, the neural subsystems involved, and the ability to monitor the progression of neural changes in the build up and resolution phases of migraine events depending on the specific type of change. Our study reveals that migraine increases the strength of perceptual centre-surround suppression for drifting stimuli at times between migraine events, when participants were asymptomatic. Further research is required to ascertain whether the strength of surround suppression varies in a systematic fashion relative to migraine event timing and to understand the underlying mechanisms for the drifting stimulus specific nature of our findings.
